# Softening of PEO–LiTFSI/LLZTO Composite Polymer Electrolytes for Solid-State Batteries under Cyclic Compression

**DOI:** 10.1021/acsaem.3c01357

**Published:** 2023-09-09

**Authors:** Dan-il Yoon, Nishad Mulay, Jericko Baltazar, Dang Khoa Cao, Valeria Perez, Johanna Nelson Weker, Min Hwan Lee, Robert D. Miller, Dahyun Oh, Sang-Joon John Lee

**Affiliations:** † Department of Chemical and Materials Engineering, San Jose State University, One Washington Square, San Jose, California 95192-0082, United States; ‡ Department of Mechanical Engineering, San Jose State University, One Washington Square, San Jose, California 95192-0087, United States; § Stanford Synchrotron Radiation Lightsource, SLAC National Accelerator Laboratory, 2575 Sand Hill Road, Menlo Park, California 94025, United States; ∥ Department of Mechanical Engineering, University of California, Merced, 5200 Lake Road, Merced, California 95340, United States; ⊥ Department of Materials Science and Engineering, Stanford University, 496 Lomita Mall, Stanford, California 94305, United States

**Keywords:** composite polymer electrolyte, cyclic compression, fatigue softening, elastic modulus, ionic conductivity, particle distribution

## Abstract

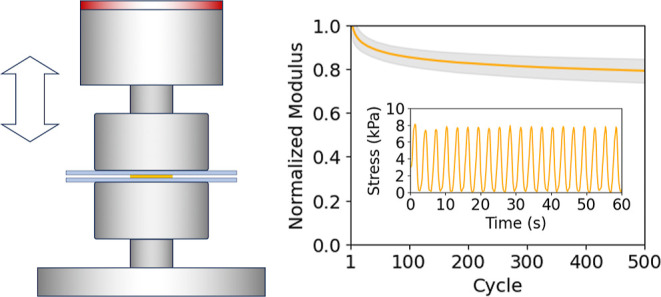

Composite polymer electrolytes (CPEs) strike an effective balance between ionic conductivity and mechanical flexibility for lithium-ion solid-state batteries. Long-term performance, however, is limited by capacity fading after hundreds of charge and discharge cycles. The causes of performance degradation include multiple contributing factors such as dendrite formation, physicochemical changes in electrolytes, and structural remodeling of porous electrodes. Among the many factors that contribute to performance degradation, the effect of stress specifically on the composite electrolyte is not well understood. This study examines the mechanical changes in a poly(ethylene oxide) electrolyte with bis(trifluoromethane) sulfonimide. Two different sizes of Li_6.4_La_3_Zr_1.4_Ta_0.6_O_12_ particles (500 nm and 5 μm) are compared to evaluate the effect of the surface-to-volume ratio of the ion-conducting fillers within the composite. Cyclic compression was applied to mimic stress cycling in the electrolyte, which would be caused by asymmetric volume changes that occur during charging and discharging cycles. The electrolytes exhibited fatigue softening, whereby the compressive modulus gradually decreased with an increase in the number of cycles. When the electrolyte was tested for 500 cycles at 30% compressive strain, the compressive modulus of the electrolyte was reduced to approximately 80% of the modulus before cycling. While the extent of softening was similar regardless of particle size, CPEs with 500 nm particles exhibited a significant reduction in ionic conductivity after cyclic compression (1.4 × 10^–7^ ± 2.3 × 10^–8^ vs 1.1 × 10^–7^ ± 2.0 × 10^–8^ S/cm, mean ± standard deviation, *n* = 4), whereas there was no significant change in ionic conductivity for CPEs with 5 μm particles. These observations —performed deliberately in the absence of charge–discharge cycles —show that repetitive mechanical stresses can play a significant role in altering the performance of CPEs, thereby revealing another possible mechanism for performance degradation in all-solid-state batteries.

## 1 Introduction

Solid electrolytes in batteries are safer than the more common organic liquid electrolytes, which are subject to concerns such as flammability and overheating.^1–3^ Poly(ethylene oxide) (PEO), which complexes with lithium salts, is one of the most widely considered materials for polymer electrolytes.^4–6^ Solid polymers have favorable toughness but suffer from limited ionic conductivity. Accordingly, composite polymer electrolytes (CPEs) incorporate filler particles for enhancing ionic conductivity.^7–10^ Active fillers with lithium, such as Li_6.4_La_3_Zr_1.4_Ta_0.6_O_12_ (LLZTO) and Li_6.4_La_3_Zr_1.4_O_12_ (LLZO), have been among the most widely investigated in recent years.^11–15^ Despite rapid advances in the development of CPEs, attention regarding the effects of compressive stress has predominantly been from the perspective of porous electrodes,^16^ electrode–electrolyte interfaces,^17^ or combined effects throughout an entire cell.^18–20^ Much less is known about the mechanical behavior of composite polymer electrolytes, particularly for long-term cycling. Stress distribution within a composite electrolyte can result in a variety of failure modes, including detachment or delamination between active materials and surrounding polymer electrolytes.^21^ Loss of contact area can occur with insufficient pressure,^22^ and the problem would be exacerbated by softening of the electrolyte. Changes in stress magnitude in solid-state batteries can be on the order of megapascals,^23,24^ and when also considering temperature increases and thermal swelling at higher charging rates, the internal strain on the battery can reach up to 15%.^25^ As battery technology continues to move toward faster charging speeds, these internal stresses and strains will play an increasingly important role in the health and performance of all-solid-state batteries.

In this study, we focus on identifying the effect of long-term mechanical cycling (500 cycles) on the CPEs, without complex material changes induced by electrochemical cycling. This approach enables us to decouple the changes in mechanical behavior from electrochemical reactions and thereby overcomes the confounding effects caused by changes at the electrode–electrolyte interface over long testing durations. By mechanically simulating the cyclic stresses potentially generated during battery cycling, we are able to evaluate the effect of long-term operation in a much shorter time (less than 1 h) than full electrochemical cycling (∼1000 h at a rate of 1 C). Using PEO–LiTFSI with LLZTO as a representative CPE, the main questions examined in this study are (1) how does the modulus change after many compressive cycles? (2) Does the particle size affect the way in which the modulus changes? (3) To what extent does cyclic compression affect ionic conductivity? Composite polymer materials and processes have been developed to achieve thermal, electrochemical, and oxidation stability,^26^ and our investigation complements such work by directing specific attention to *mechanical* stability.

## 2 Methods

### 2.1 Specimen Fabrication

PEO–LiTFSI electrolytes were fabricated by solution casting with the addition of LLZTO powder (Ampcera, Inc., Milpitas, California, USA), PEO (*M*_v_ = 600,000 g/mol, Sigma-Aldrich Corp., St. Louis, Missouri, USA), and LiTFSI (Gotion, Inc., Fremont, California, USA). For the LLZTO, two different mean particle sizes were compared, 500 nm and 5 μm. Solutions were prepared with a 43:1 EO/Li molar ratio, and LLZTO was included at a concentration of 24 wt %. All three components (PEO, LiTFSI, and LLZTO) were dried in a vacuum oven for a minimum of 24 h at 60 °C. After drying, they were transferred to and kept inside an argon-gas glovebox. Solutions were prepared by first mixing LiTFSI, PEO, and LLZTO in anhydrous acetonitrile (ACN, Sigma-Aldrich) at room temperature for 24 h. The mixed solution was cast onto a flat polytetrafluoroethylene (PTFE) plate using a doctor blade with a 1.2 mm gap. Casting was performed in a nitrogen-purged bag. The cast films were dried in a vacuum oven for a minimum of 24 h at 60 °C. After drying, the freestanding film was peeled from the plate and punched into circular disks using a 15.9 mm diameter punch. The punched specimens (∼50 μm thick) were kept in nitrogen storage prior to testing.

### 2.2 Mechanical Compression

**Figure 1:**
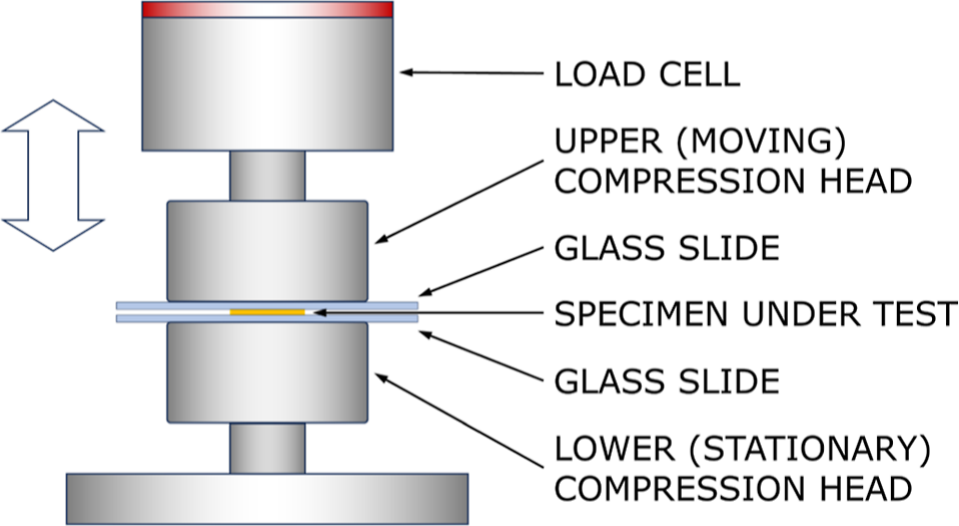
Configuration for mechanical compression testing.

All mechanical tests were performed using an Instron ElectroPuls E1000 dynamic testing system (Instron, Norwood, Massachusetts, USA) with stainless steel compression heads (Figure 1). Compressive strain was prescribed according to the measured thickness of each CPE specimen, and force was measured by an inline load cell at a sampling rate of 5 Hz. Film thickness for each test specimen (nominally ∼50 μm) was measured immediately before and after cycling by placing the material between two borosilicate glass slides and measuring to the nearest 1 μm with a digital micrometer. The load cell is rated as having 40 μm at full-scale (250 N), such that at typical maximum loads (∼2 N), the maximum parasitic deflection is ∼0.3 μm.

For each particle size, the average response of *n* = 4 replicates was measured to quantify specimen-to-specimen variability. Stress–strain curves were fit based on ten forward–compression cycles, using an exponential function of the form σ = *C*(exp(*k*ε) – 1), where σ is the compressive stress, ε is the axial strain, and *C* and *k* are fitting parameters. Cyclic compression was applied to 30% strain in 500 cycles, with the force measurements converted to secant modulus *E*_s_^*^ (at the maximum strain). The secant modulus is pragmatically convenient because when normalizing to the maximum value, the secant modulus is equivalent to the normalized stress at the given strain. Softening behavior is fit to a power-law model of the form *E*_s_^*^ = *A*(*N*)^*b*^, where *N* is the number of cycles and *A* and *b* are dimensionless fitting parameters. For all mechanical tests, a small preload was used to ensure consistent contact with the specimen before each cycling run begins. This preload was selected to be 70 mN, corresponding to less than 5% of the typical peak stresses (10 kPa) observed during setup trials. Data analysis and curve fits were performed using Python, using raw data exported from the Instron interface software (WaveMatrix 1.8). The SciPy^27^ library for Python was used for curve fitting, and quality of fit is determined by root-mean-square error (RMSE) between raw data points and the fitted curve. The Python statistics library^28^ was used for statistical analysis, and a significance level of α = 0.05 was used as the threshold for statistical significance.

### 2.3 Imaging and Topography Mapping

Uniform particle dispersion without agglomeration is important for maximizing the efficiency of interfacial regions between ion-conducting particles and their surrounding polymer matrix.^29^ Clustering of particles is also known to affect the overall Young’s modulus for nanoreinforced polymer composites.^30^ In order to examine particle distribution, transmission X-ray microscopy (TXM) was performed for both particle sizes (500 nm and 5 μm), examining the as-fabricated specimens as well as those that were subjected to cyclic compression. A circular specimen of 15.9 mm diameter and a nominal thickness of 40 μm (±15 μm) was cut into triangular sections to create an apex with a ∼60° angle as the target for tomographic imaging. Using beamline 6-2c at the Stanford Synchrotron Radiation Lightsource (SSRL), images were acquired at 8355 eV with a pixel size of 35.2 nm. The images at each angle were acquired with a 3 × 2 mosaic at every 1° increment for 180° as the specimen was rotated with 30% overlap of mosaic tiles. This method allows for a final 3-D image over an area that is larger than the nominal field of view (∼33 μm × ∼33 μm; 1024 × 1024 pixels) and resulted in the creation of 181 image files of 2470 × 1743 pixels. Ten reference images were captured and averaged every 45°. Data processing was performed using the TXM Wizard software,^31^ which involved reference correction followed by aligning and stitching of the mosaic tiles. Tomographic reconstruction was also performed using TXM Wizard 20 iterations of the algebraic reconstruction technique (ART) after manual alignment of the mosaicked projection images. The reconstructed volumes have a resolution of approximately 60 nm. Dragonfly software (Object Research Systems, Montréal, Québec, Canada) was used to create 3-D renderings for each reconstructed volume. Scanning electron microscopy (SEM) was performed using an FEI Quanta 200 scanning electron microscope.

Significant changes in topography can affect the low-strain response as the undulations are flattened. Surface roughness also determines true contact area and can affect interfacial performance in working batteries.^32^ Irregularities and inconsistencies in surface topography decrease the contact surface and directly affect the contact pressure of the CPEs. Although model fitting in impedance spectroscopy distinguishes bulk resistance from interfacial resistance, the discrepancies in the actual contact area during measurements can affect the corresponding computed values of *resistivity* and (by inverse) the reported values of ionic conductivity. Accordingly, quantitative measurements of surface topography were made to check whether repetitive compression might have been substantial enough to alter the geometric interface between the characteristically undulated PEO–LiTFSI surfaces and mating contacts (e.g., stainless steel disks used as blocking electrodes during impedance measurements). For these topography measurements, scanning white light interferometry (Wyko NT9100, Bruker, Camarillo, California, USA) was used to extract the height profiles of the surface of the representative PEO–LiTFSI specimens before and after the cyclic compression tests. Measurements were taken at two locations approximately 0.2 mm on each side of a laser-scribed fiducial mark, where the fiducial mark ensures that the same locations are examined before and after cyclic compression. For each 3-D scan, surface roughness was quantified by exporting the raw scan data as XYZ coordinates and using topography analysis software (ProfilmOnline, Filmetrics, San Diego, California, USA) to compute the RMS profile.

### 2.4 Differential Scanning Calorimetry

Polymer electrolytes exhibit high ionic conductivity with low degrees of crystallinity as conduction is favorable in an amorphous state.^33,34^ To examine crystallinity, thermal analysis was performed by differential scanning calorimetry (DSC) using a differential scanning calorimeter (Q20, TA Instruments New Castle, Delaware, USA). Normalized endothermic enthalpy and peak temperature were measured for a compressively cycled sample and compared to those of a control sample from the same fabrication batch that was not subjected to compression. Measurements were performed using 5 mg of material samples over a temperature range from 30 to 200 °C, with a heating rate of 10 °C/min and nitrogen as the purging gas. Two consecutive heating cycles were used for the DSC measurements. The first heating cycle was used to eliminate residual contaminants or moisture present in the specimens or aluminum sample pans. The melting enthalpy (Δ*H*_m_) and melting temperature (*T*_m_) of the specimens were determined from the endothermic peak of the second heating cycle. Crystallinity χ_c_ for PEO–LiTFSI was calculated from normalized enthalpy according to the formula χ_c_ = Δ*H*_m_/(Δ*H*_PEO_·*f*_PEO_),^26^ where Δ*H*_PEO_ is the enthalpy of fusion for fully crystalline PEO (203 J/g)^35^ and *f*_PEO_ is the weight fraction of PEO (0.87 for the 43:1 EO/Li specimens as prepared).

### 2.5 Ionic Conductivity

Impedance was measured with an Interface 1010E (Gamry Instruments, Warminster, Pennsylvania, USA), from 2 MHz to 0.1 Hz with 10 mV AC voltage. Each electrolyte specimen was tested in a CR 2032 coin-cell holder between 15.5 mm diameter stainless steel disks (MTI Corporation, Richmond, California, USA) that served as ion-blocking electrodes. Except for the minimum time necessary for mechanical testing or EIS measurements, all CPEs were kept in nitrogen-purged storage. An equivalent circuit model R1–(R2/Q2)–Q3 was used for fitting of impedance measurements and determination of bulk resistance using impedance analysis software (Zfit from BioLogic, Seyssinet-Pariset, France). *R*_1_ represents the contact resistance; *R*_2_ and *Q*_2_ represent the bulk resistance and bulk capacitance of the electrolyte, respectively; and *Q*_3_ represents the capacitance between the electrolyte and the stainless steel spacers. Ionic conductivity, κ, is calculated from bulk resistance *R*_b_ = *R*_2_ according to the formula κ = *h*/(*R*_b_*A*), where *h* is the thickness of the electrolyte and *A* is the cross-sectional area.

## 3 Results and Discussion

### 3.1 Stress–Strain Response

**Figure 2:**
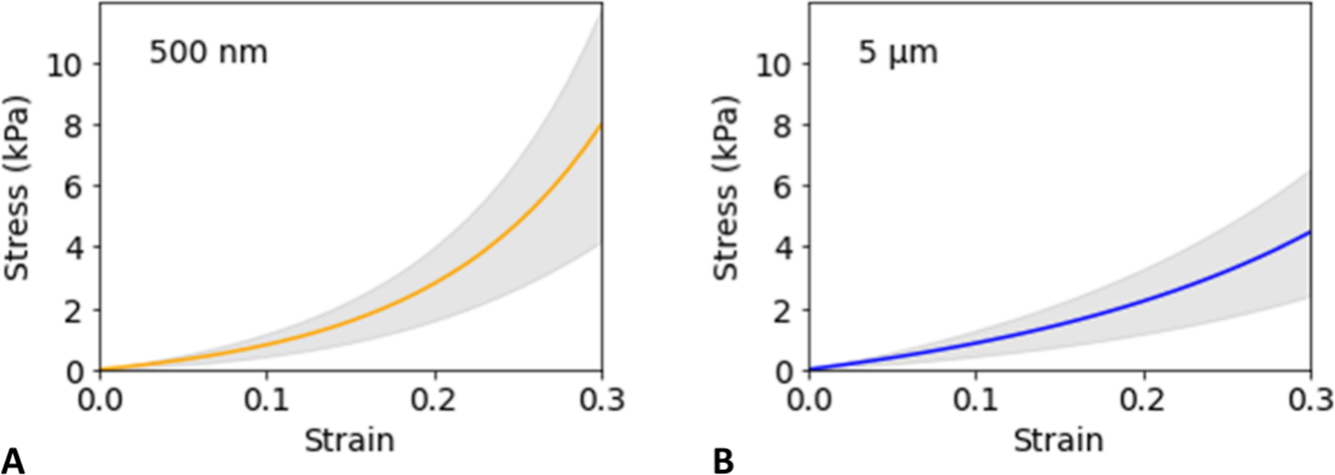
Stress–strain curves for PEO–LiTFSI electrolytes with 500 nm LLZTO (A) and 5 μm LLZTO (B). The solid curves show exponential fits to the experimental averages at each cycle number, and the shaded regions indicate standard deviation among the *n* = 4 identically prepared replicates for each respective case. The raw data and details regarding curve fitting and quality-of-fit are provided in the Supporting Information.

Figure 2 compares the stress–strain curves for the specimens fabricated with 500 nm and 5 μm LLZTO. CPEs with the smaller particle size exhibited more pronounced strain stiffening (i.e., steeper slope at large strains compared to small strains). At the maximum strain of 30%, the secant modulus *E*_s_ (i.e., the slope of a straight line from the origin to a specific point along the stress–strain curve) was 79% higher than that for the smaller particle size (average and standard deviation of 26.6 ± 12.7 kPa for 500 nm LLZTO vs 14.9 ± 6.88 kPa for 5 μm LLZTO).

A review of several polymer matrix composites with different filler particle materials reported that the Young’s moduli of many composites are relatively insensitive to particle sizes above ∼30 nm.^36^ However, Young’s modulus is limited to the linear regime, and indeed, in Figure 2, the stress–strain curves for both particle sizes are very similar up to a strain of 0.2. Only at higher strain does the difference in stiffness become more pronounced. A plausible reason for the higher stiffness at larger strains for the smaller particles is that the average distance between neighboring particles is smaller for smaller particles, thus achieving more spatially efficient influence over the surrounding material. SEM images (shown subsequently in Section 3.3) show a nearly uniform distribution of the 500 nm particles without severe agglomeration, where the typical distance between particles is on the order of a few microns. In contrast, the larger 5 μm particles can be tens of microns apart. The effect of interfacial adhesion energy between particles and the polymer matrix is another possible factor influencing the modulus of the composite, where smaller particles have substantially larger surface-to-volume ratio. Assuming an approximately spherical shape, the surface-to-volume ratio varies inversely proportionally to radius *r* [i.e., (4π*r*^2^)/((4/3)π*r*^3^) = 3/*r*]. Thus, for the same volume ratio, 500 nm particles would have more surface area than 5 μm particles by an order of magnitude. Although it has been observed that interfacial adhesion has limited effect on modulus,^36^ smaller particles are recognized as having an indirect effect by serving as well-distributed nucleation sites for polymer crystallization, which can in turn increase the modulus of the composite.^37^

### 3.2 Fatigue Softening

**Figure 3:**
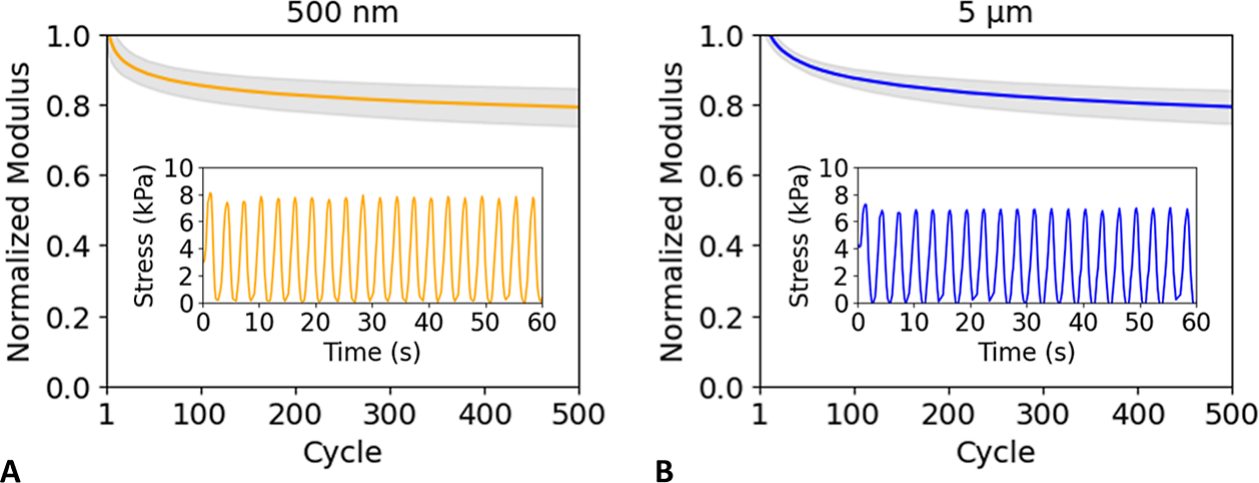
Normalized secant modulus vs number of cycles for PEO–LiTFSI electrolytes with 500 nm LLZTO (A) and 5 μm LLZTO (B). The solid curves show power law fits to the experimental averages at each cycle number, and the shaded regions indicate standard deviation among the *n* = 4 identically prepared replicates for each respective case. The insets show close-up details of stress vs time for representative individual specimens for each particle size. The raw data and details regarding curve fitting and quality-of-fit are provided in the Supporting Information.

Figure 3 shows the accumulated effect of cyclic compression over the entire duration of 500 cycles for the two different particle sizes. The power-law dependence is consistent with Basquin’s law of fatigue^38^ and progressive stress softening that is also observed in elastomers.^39^

For both particle sizes, the data show that the modulus has a steeper decline within the first ∼100 cycles, followed by continual reduction in modulus. After 500 cycles, the modulus in both cases is reduced to slightly below 80% of its original value, and with the uncertainty bands, the rate of decline is almost indistinguishable. Specifically, the average normalized moduli (*n* = 4 replicates each) of the last 50 cycles for 500 nm and 5 μm particles were 0.78 and 0.77, respectively. The standard deviation appears to be slightly larger for the 500 nm case, but otherwise, the variability among the replicates appears similar for both particle sizes. As confirmed in Figure S4, there was no significant change in specimen thickness before and after cyclic compression. The close similarity in softening behavior between CPEs with 500 nm and 5 μm particles (both at 24 wt %) suggests that softening is independent of LLZTO particle size. In order to check further if softening was affected by filler particle *presence*, a follow-up experiment was conducted for PEO–LiTFSI electrolytes with no LLZTO by applying the same cyclic compression method. The average normalized modulus (*n* = 3 replicates) of the last 50 cycles with no particles was 0.87. Thus, approximately half of the softening is attributed to the polymer itself, with an additional contribution of similar magnitude coming from the presence of filler particles. Softening of solid polymer electrolytes without particles has also been observed in cyclic compression of thick PEO–LiClO_4_ cylinders with thickness in the range of 3–6 mm.^40^ Our observation of a combined ∼20% total reduction in modulus that develops incrementally over hundreds of cycles of stress loading indicates how batteries using such composite polymer electrolytes can be more susceptible to long-term problems such as dendrite growth at the electrode interfaces.

There is no evidence to suggest that the observed softening is attributed to temperature changes. Although polymer composites under repeated deformation are susceptible in general to self-heating,^41,42^ the test specimens in these experiments were very small compared to the large steel compression heads (50 mm in diameter and 25 mm in height, shown approximately to scale in Figure 1), such that ambient temperature was maintained. To check the temperature conditions, a disk-shaped thermocouple was placed in contact with one of the steel spacers for a representative PEO–LiTFSI electrolyte. Temperature change was observed to be negligible, with fluctuations of no greater than 1 °C throughout the duration of 500 cycles at 30% strain.

### 3.3 Particle Distribution and Topography

**Figure 4:**
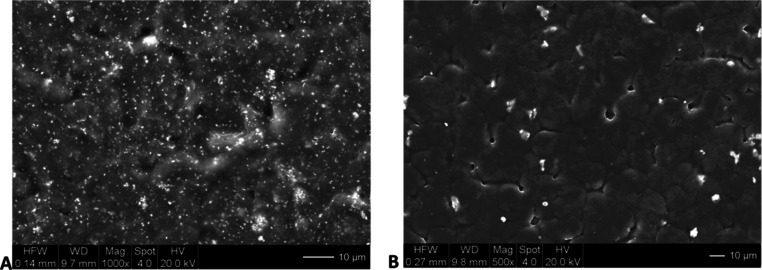
SEM images of surfaces for PEO–LiTFSI electrolytes with (A) 500 nm and (B) 5 μm LLZTO particles, where the particles appear as white spots.

Figure 4 shows the SEM images of the surface of representative CPEs with 500 nm and 5 μm LLZTO particles (both at 10 wt %). In both cases, the distribution of particles on the surface appears to be random and uniform. The wide field of view (Figure 4B) reveals some long-range surface undulations in the PEO. Both the distribution of particles and the undulated texture of the PEO are consistent with SEM imaging of similar particle-filled PEO-based electrolytes.^43^

**Figure 5:**
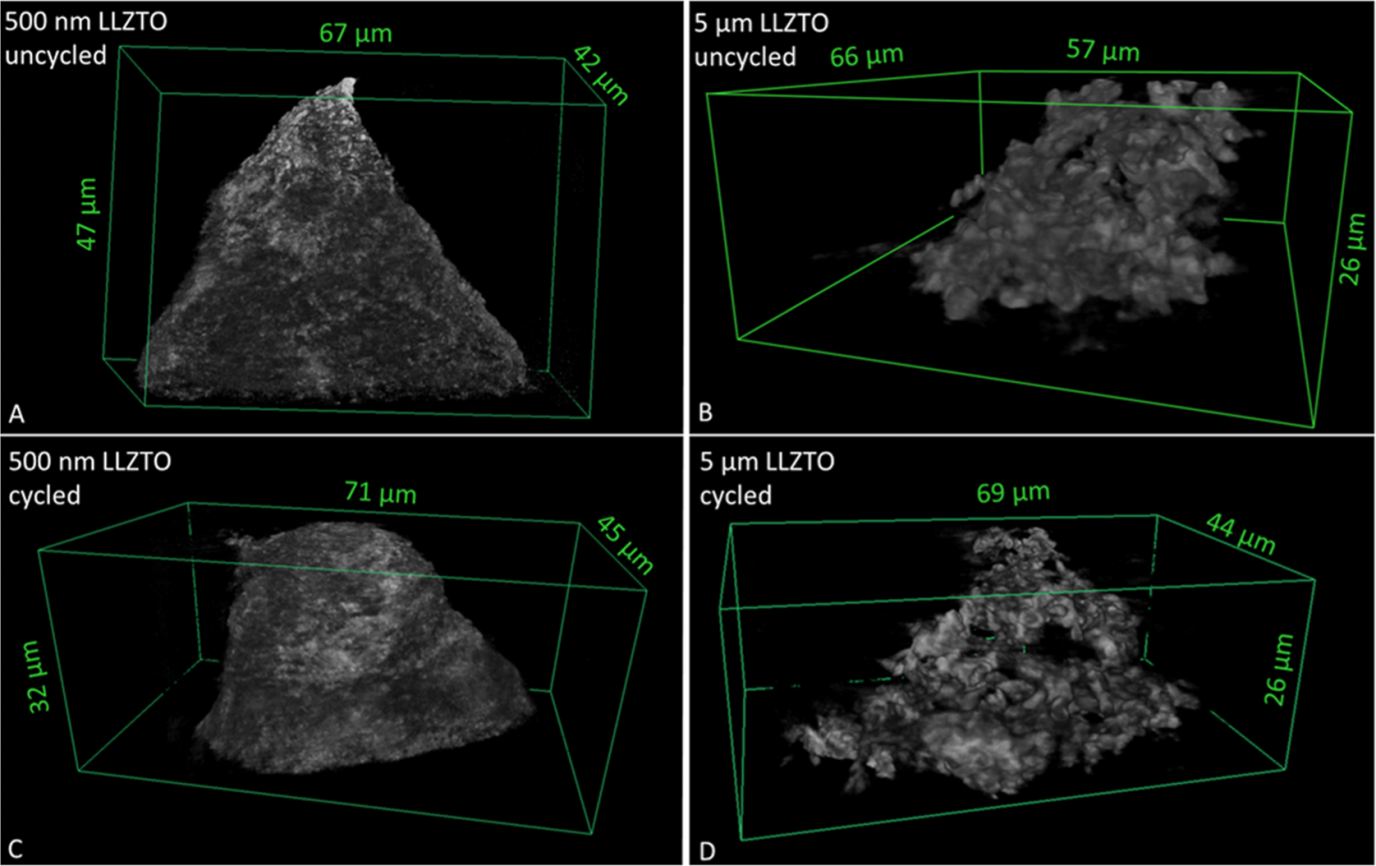
3-D X-ray tomography renderings at the apex of PEO–LiTFSI electrolytes before (A,B) and after (C,D) 500 cycles of mechanical compression.

Figure 5 shows the internal 3-D distribution of particles by X-ray tomography and also compares representative examples before and after 500 cycles of compression to 30% strain. The images are representative of the given particle sizes and concentration (500 nm or 5 μm, both at 24 wt %) but not the exact same location of the exact same specimen before and after cyclic compression. The distribution of 500 nm particles throughout the 3-D volume is overall uniform. Although the larger (5 μm) particles appear to show potential evidence of particle redistribution after cycling, the softening is very similar for both particle sizes (Figure 3), suggesting that particle redistribution is not a major factor in determining the macroscopic softening behavior of the CPEs.

**Figure 6:**
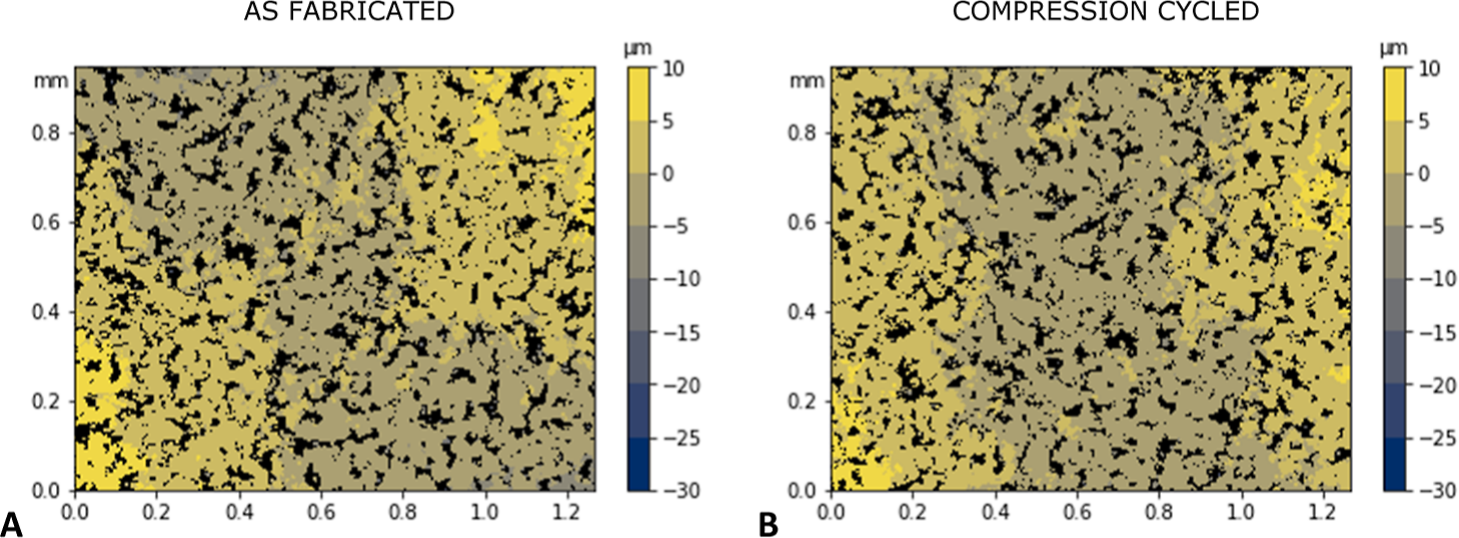
Topography map of representative sites on a representative PEO–LiTFSI electrolyte as-fabricated (A) and compression cycled (B), where laser-scribed fiducial marks were used to register the location of the same field of view.

Figure 6 shows topographical maps for a representative PEO–LiTFSI electrolyte without LLZTO, before (Figure 6A) and after (Figure 6B) 500 cycles of compression to 30% strain. Consistent with what is seen in the SEM images (Figure 4), the undulations of the PEO–LiTFSI are on the order of tens of micrometers, substantially larger than LLZTO particles. The RMS surface roughness of the CPEs decreased slightly from the as-fabricated value of 2.59 μm to 2.17 μm after mechanical cycling (averaged using two independent specimens and two distinct sites for each). The absence of discernible differences before and after cyclic compression in Figure 6 shows that the observed softening of the material is likely to be a characteristic of the structural changes within the bulk rather than on the surface of the material. Significant change in topography would also be a concern because it can be a contributing factor to high ionic conductivity by increasing the true contact area against flat electrodes used in EIS measurements. However, the difference in surface roughness is (by nature of the contactless optical measurement) in the free state, and even under slight compression, it is expected that the difference would be even smaller. Thus, this observation that surface roughness has not changed substantially provides evidence that geometric contact area is a major contributing factor to the observed changes in ionic conductivity before and after cyclic compression.

### 3.4 Crystallinity

**Figure 7:**
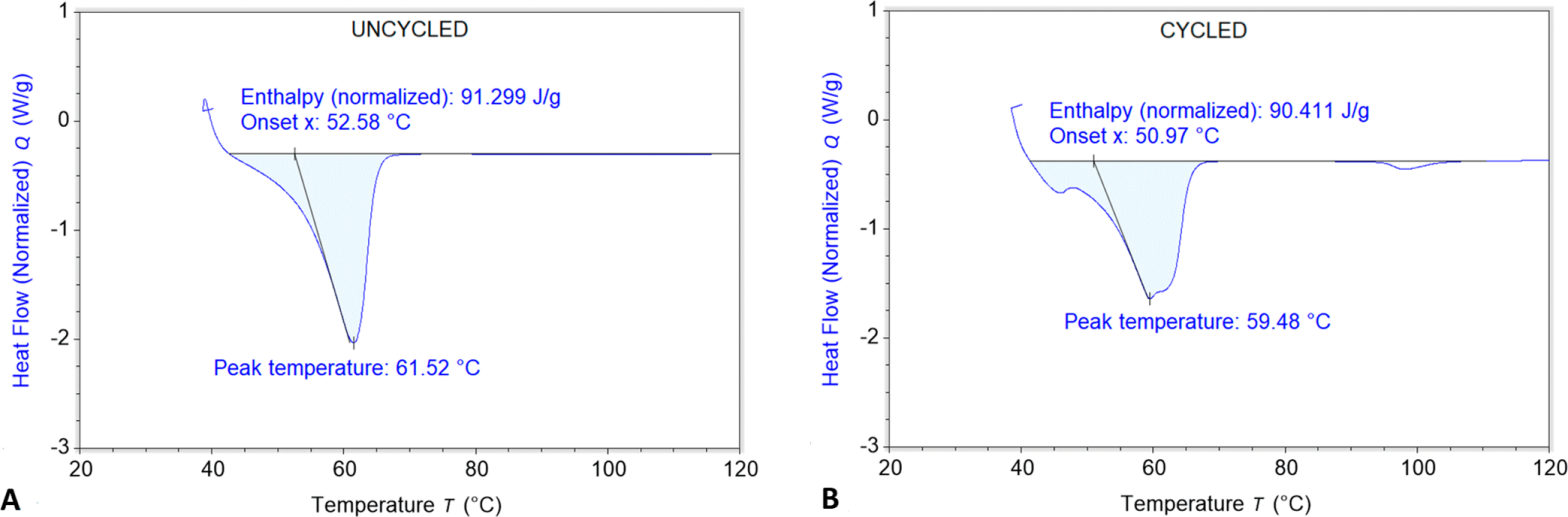
Thermograms of PEO–LiTFSI before (A) and after (B) 500 cycles of compression to 30% strain.

Figure 7 shows contrasting DSC thermograms of PEO–LiTFSI before and after 500 cycles of compression to 30% strain. The enthalpy and peak temperatures were similar in both cases, with corresponding crystallinity values of 51.8% before and 51.3% after cyclic compression. Although bulk crystallinity is similar, the cycled case reveals broadening and a secondary peak, showing potential evidence of a change in the microstructure (e.g., spatial redistribution of crystalline and amorphous regions).

There is a substantial difference in the elastic modulus between crystalline and amorphous PEO, and under sufficiently high local strain, it is possible that the spatial distribution of spherulites can change. The stiffness of PEO (even with the same molecular weight) can vary by more than an order of magnitude, and the elastic modulus is correlated with the extent of crystallinity. For example, PEO (400k g/mol) was determined by DSC to be 96% crystalline and had an elastic modulus of 333 MPa, whereas PEO with LiTFSI was 51% crystalline with an elastic modulus of 23.2 MPa.^44^ For tensile loading of PEO-based electrolyte films that were stretched beyond 50% strain, it has been observed under SEM imaging that amorphous regions can experience local elongation beyond yield limits.^40^ Although smaller in magnitude, local strains under compression in the through-plane direction may similarly cause subtle rearrangement of the semicrystalline architecture, especially when accumulated over hundreds of cycles. Compression of other semicrystalline polymers (e.g., polyethylene) is capable of altering crystallinity by a combination of mechanisms that can include interlamellar sliding, crystallographic slip, transverse slip, and/or chain slip.^45^ Such remodeling of the PEO–LiTFSI microstructure, exacerbated by the presence of particles, offers a plausible reason for the softening behavior observed in our cyclic compression loading (Figure 3) and reveals an otherwise subtle degradation mechanism for solid polymer electrolytes in rechargeable batteries.

### 3.5 Ionic Conductivity

**Figure 8:**
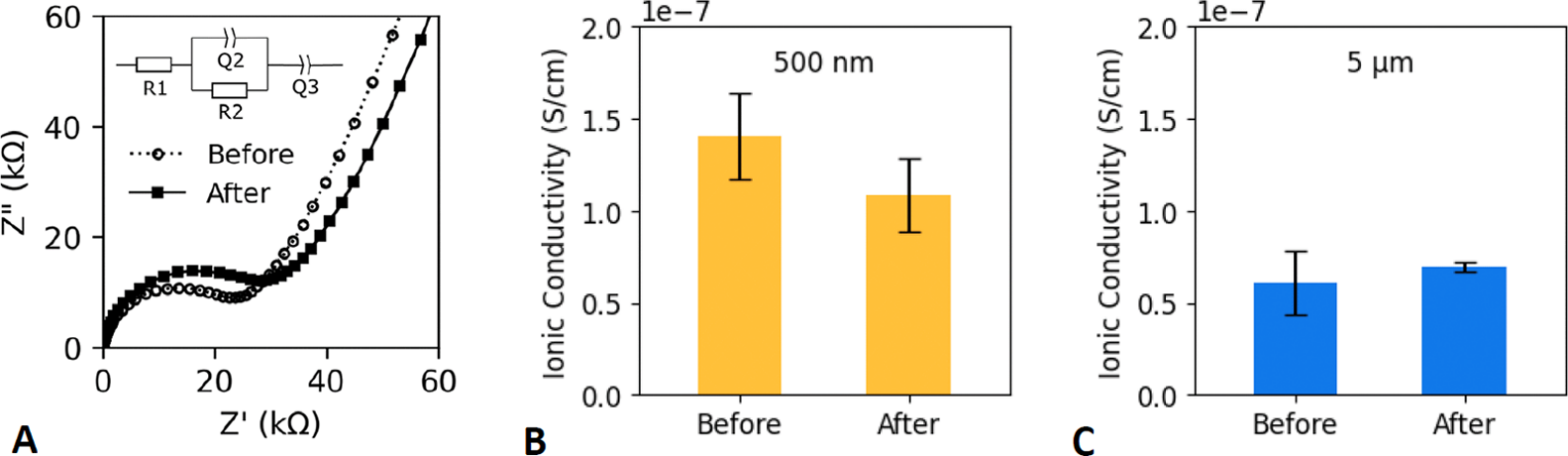
Representative EIS curves for a representative electrolyte (with 500 nm LLZTO particles) before and after cyclic compression (A) with the equivalent circuit (inset) used to quantify the ionic conductivity. Ionic conductivity comparison before and after 500 cycles of compression to 30% strain, for electrolytes with 500 nm LLZTO particles (B) and ionic conductivity comparison likewise for 5 μm LLZTO particles (C). Column height represents mean values for *n* = 4 replicates, and error bars indicate the corresponding standard deviations.

Figure 8 compares the measured values of ionic conductivity for the two different particle sizes before and after 500 cycles of applied compression. We chose two different sizes of LLZTO particles, 500 nm and 5 μm, to elucidate the effect of specific surface area (between particles and polymer) on lithium conduction. CPEs with 500 nm particles (Figure 8B) exhibit a 23% reduction in average ionic conductivity after cycling (1.4 × 10^–7^ ± 2.3 × 10^–8^ vs 1.1 × 10^–7^ ± 2.0 × 10^–8^ S/cm, mean ± standard deviation), and the difference based on a one-sided *t*-test assuming unequal variances is statistically significant (*p* = 0.042). Ionic conductivity has been observed to change based on tensile strain applied to PEO electrolytes,^46^ and irreversible chain detachment and decomposition have been identified as a major contributor to fatigue softening of elastomers.^39^ To our knowledge, similar studies have not been reported for cyclic mechanical compression of CPEs. CPEs with 5 μm particles (Figure 8C) exhibited lower ionic conductivity than that of the 500 nm case. There is a slight increase in average ionic conductivity for the 5 μm values after cycling (6.1 × 10^–8^ ± 1.7 × 10^–8^ vs 6.9 × 10^–8^ ± 2.6 × 10^–9^ S/cm), although the difference is not statistically significant (*p* = 0.21). Interestingly, cyclic compression reduced the standard deviation among replicates for the 5 μm particles, suggesting that mechanical preconditioning of the material can potentially reduce variability for CPEs with larger particles. Although for PEO–LiTFSI with low molecular weight (*M*_n_ = 550 g/mol), rheological measurements have shown that conductivity tends to decrease with increasing *shear* modulus,^47^ among these 16 measurements with much higher molecular weight (*M*_v_ = 600,000 g/mol), no strong correlation was observed between ionic conductivity and compressive modulus. A correlation plot is included in the Supporting Information (Figure S3).

While similar findings showing higher conductivity for smaller particles have been reported for other PEO-based composite electrolytes,^48–50^ these data uniquely show a difference in the sensitivity of ionic conductivity to particle size, when subjected to repetitive mechanical deformation. That is, the smaller particles are associated with higher ionic conductivity but are also more prone to proportional loss of conductivity when subjected to repetitive mechanical loading. By adding this new perspective of loading history and its effect on modifying mechanical behavior, these findings can complement atomistic modeling of the role of particle size on the dynamics of polymer chains, where smaller particles are deemed more effective in enhancing lithium-ion mobility.^51^ There are multiple theories regarding which of the pathways for lithium-ion transport (i.e., through the polymer, through the ceramic, or along the interfaces between the two phases) dominates ionic conductivity.^52^ Given that the interfacial surface area for 500 nm particles is larger than that for 5 μm particles by an order of magnitude and that there correspondingly are more particle–polymer interfaces per unit volume, we believe that the larger difference in ionic conductivities with 500 nm particles highlights the significant role of the ceramic–polymer interface in ion conduction after mechanical cycling. The lower ionic conductivity of CPEs with 500 nm particles after cyclic compression thus reveals a potential negative impact on the interfacial lithium-ion conduction pathways caused by mechanical cycling. Nuclear magnetic resonance (NMR) methods have shown that smaller particles (30 vs 200 nm) are associated with looser chain folding structure based on characteristic relaxation times of polycaprolactone (PCL) electrolytes with LiTFSI and Al_2_O_3_ particles.^53^ Alteration of chain folding over many cycles of structural deformation can have an adverse effect on lithium-ion transport through the electrolyte in a way that is more pronounced for composites with smaller particles.

## 4 Conclusions

In this work, we have shown how cyclic compression of a CPE results in fatigue softening of the material decoupled from any complex electrochemical reactions. First, we observed that the composite electrolytes exhibit a fatigue softening behavior in which the compressive modulus was reduced to approximately 80% of its value before mechanical cycling. It was additionally revealed that the LLZTO particle size has little impact on the fatigue softening behavior. Interestingly, however, composite electrolytes with 500 nm LLZTO particles exhibited a substantial (23%) reduction in ionic conductivity after 500 cycles of compression to 30% strain. At the same weight fraction (24 wt %) in PEO–LiTFSI, CPEs with 5 μm particles had lower ionic conductivity by approximately a factor of 2, although— in contrast to the 500 nm case, —the conductivity was hardly affected by cyclic compression. X-ray tomography and optical profilometry measurements suggest that the softening is not dominated by the changes in particle distribution or surface roughness, while DSC thermograms and physical argument suggest that the observed softening is driven by changes to the semicrystalline microstructure. In CPE battery assemblies, the modulus of the electrolyte affects the overall stress state and has relevance in important concerns such as dendrite suppression and electrode damage. The modulus of CPEs, including consistency and stability thereof, is important for long-term high performance of all-solid-state batteries. Fatigue softening in electrolytes can lead to a weaker contact pressure at interfaces between the cathode, anode, and electrolyte, resulting in diminished and less consistent battery performance. Useful battery life can vary depending on the cathode and charging rates used, but most lithium-ion batteries generally have a useful lifetime from several 100 to over 1000 cycles.^54^ Therefore, cyclic compression testing as presented in this paper can offer an accelerated analogue to the stress changes experienced by the polymer electrolyte over the functional lifetime of a battery. Our observation of fatigue softening may also motivate deliberate mechanical preconditioning by cyclic compression as a value-added manufacturing step in order to establish more consistent material behavior and morepredictable long-term battery performance.

## Supporting Information Available

Additional experimental details, materials, and methods, raw stress–strain data and curve fitting, raw fatigue softening data and curve fitting, ionic conductivity vs compressive modulus, and thickness before and after cyclic compression (PDF)
3-D video renderings of particle distribution (500 nm, uncycled) in X-ray tomography scans (AVI)
3-D video renderings of particle distribution (500 nm, cycled) in X-ray tomography scans (AVI)
3-D video renderings of particle distribution (5 μm, uncycled) in X-ray tomography scans (AVI)
3-D video renderings of particle distribution (5 μm, cycled) in X-ray tomography scans (AVI)

## Supplementary Material

ae3c01357_si_001.pdf

ae3c01357_si_002.avi

ae3c01357_si_003.avi

ae3c01357_si_004.avi

ae3c01357_si_005.avi
