# Supplementary material for: Softening of PEO–LiTFSI/LLZTO Composite Polymer Electrolytes for Solid-State Batteries under Cyclic Compression
Source: ACS Appl Energy Mater. 2023 Sep 9;6(18):9400–8. doi: 10.1021/acsaem.3c01357 (PMC10538521; doi:10.1021/acsaem.3c01357)
Supplement: Supplementary file 1 — ae3c01357_si_001.pdf [file ae3c01357_si_001.pdf]

# Supporting Information

# Softening of PEO-LiTFSI/LLZTO Composite Polymer Electrolytes for Solid-State Batteries under Cyclic Compression

*Dan-il Yoon [a], Nishad Mulay [b], Jericko Baltazar [b], Dang Khoa Cao [b], Valeria Perez [a], Johanna Nelson Weker [c], Min Hwan Lee [d], Robert D. Miller [e], Dahyun Oh [a *], Sang-Joon John Lee [b *]*

*[a] Department of Chemical and Materials Engineering, San Jose State University, One Washington Square, San Jose, CA 95192-0082 USA*

*[b] Department of Mechanical Engineering, San Jose State University, One Washington Square, San Jose, CA 95192-0087 USA*

*[c] Stanford Synchrotron Radiation Lightsource, SLAC National Accelerator Laboratory, 2575 Sand Hill Road, Menlo Park, CA, 94025 USA*

*[d] Department of Mechanical Engineering, University of California, 5200 Lake Road, Merced, CA 95340 USA*

*[e] Department of Materials Science and Engineering, Stanford University, 496 Lomita Mall, Stanford, CA 94305 USA*

** Corresponding authors:*

1. Sang-Joon John Lee sang-joon.lee@sjsu.edu
2. Dahyun Oh dahyun.oh@sjsu.edu

**Raw Stress-Strain Data and Curve Fitting**

Figure S1 shows the raw stress values during applied compression to 30% strain for the PEO-LiTFSI electrolytes with 500 nm and 5 µm LLZTO for $n = 4$ replicates in each case. Separately for the two different particle sizes, each color represents a distinct replicate. When fitting stress-strain curves (as shown in Figure 1 in the main text), cycles #2 through #11 of compression were used as representative of the initial elastic behavior for each specimen. Cycle #1 was not included because it is typically subject to first-contact irregularities, such as flattening of asperities.

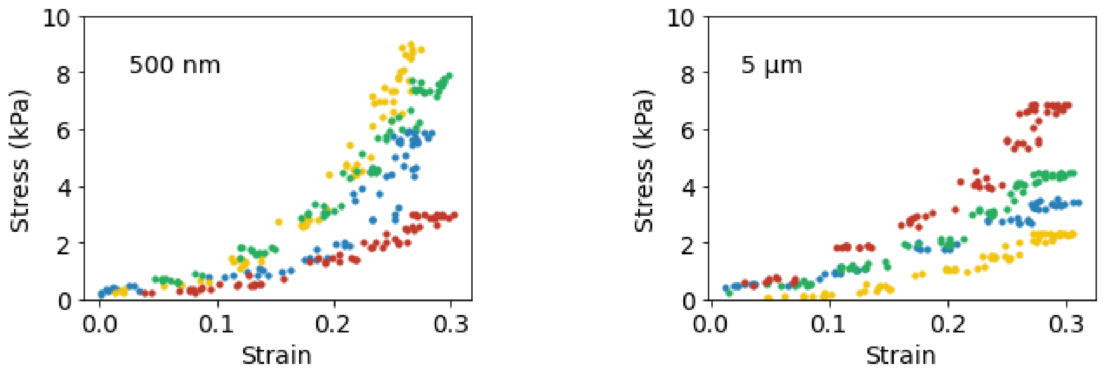


**Figure S1.** Raw stress-strain data for PEO-LiTFSI electrolytes with 500 nm (left) and 5 µm LLZTO (right).

Table S1 shows the fitting parameter values for the curves displayed in Figure 1 of the main body text. The stress-strain are fit curves to an exponential function of the form $\sigma = C(\exp(k\varepsilon) - 1)$, where $\sigma$ is the compressive stress, $\varepsilon$ is the axial strain, and $C$ and $k$ are fitting parameters. Quality of fit is quantified by root mean square error (RMSE) between raw data points and the fitted curve.

**Table S1. Fitting parameters for stress-strain curves.**

| | 500 nm LLZTO | | | 5 μm LLZTO | | |
|---|---|---|---|---|---|---|
| Replicate | *C* (kPa) | *k* | RMSE (kPa) | *C* (kPa) | *k* | RMSE (kPa) |
| 1 | 0.230 | 11.9 | 0.621 | 3.51 | 2.29 | 0.191 |
| 2 | 0.511 | 10.8 | 0.485 | 0.251 | 7.91 | 0.161 |
| 3 | 1.27 | 6.76 | 0.410 | 1.32 | 5.07 | 0.242 |
| 4 | 0.467 | 6.86 | 0.181 | 2.01 | 5.08 | 0.404 |

## Raw Fatigue Softening Data and Curve Fitting

Figure S2 shows stress magnitudes over the full duration of 500 cycles. The maximum strain was uniformly 30% at each cycle, such that trends in peak stress are also indicative of trends in compressive modulus. All specimens exhibit fatigue softening, whereby the peak stress (and likewise the secant modulus) decreases with an increasing number of cycles.

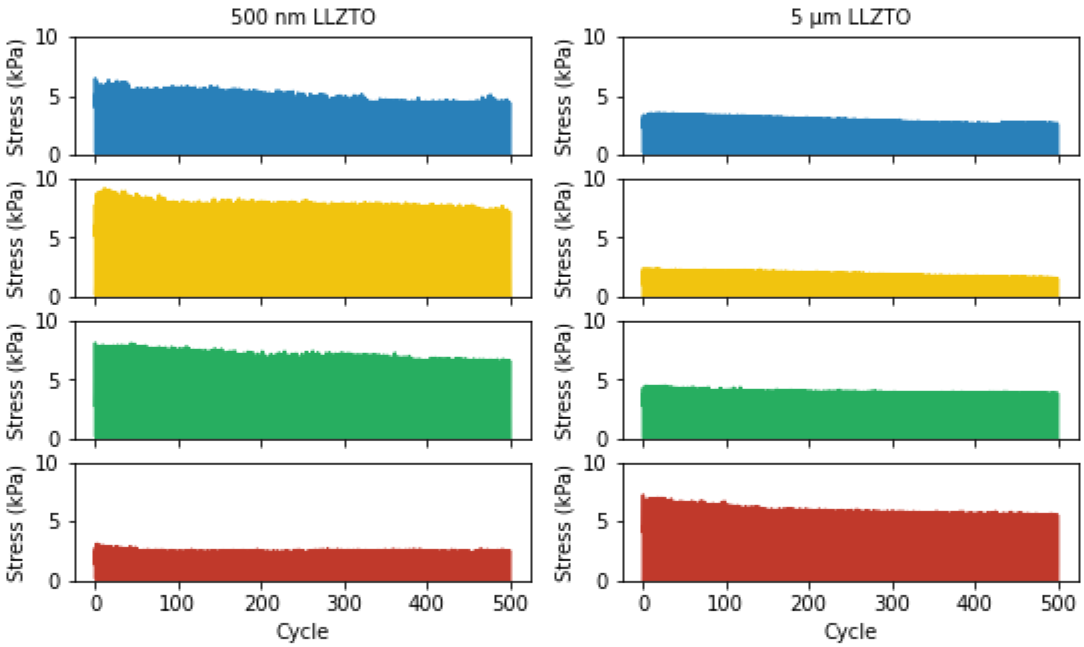


**Figure S2.** Stress vs. cycles for $n = 4$ replicates each of PEO-LiTFSI polymer electrolytes with 500 nm LLZTO (left) and 5 µm LLZTO (right).

Table S2 shows the fitting parameters for normalized secant modulus $E_s^*$ vs. number of cycles $N$, as displayed in Figure 2 of the main body test. The fits are made to a power-law model of the form $E_s^* = A(N)^b$, where $A$ and $b$ are fitting parameters, and quality of fit is quantified by RMSE.

**Table S2. Fitting parameters for fatigue softening curves.**

| | 500 nm LLZTO | | | 5 µm LLZTO | | |
|---|---|---|---|---|---|---|
| Replicate | *A* | *b* | RMSE | *A* | *b* | RMSE |
| 1 | 1.17 | -0.0783 | 0.0452 | 1.23 | -0.0726 | 0.0457 |
| 2 | 1.04 | -0.0362 | 0.0228 | 1.25 | -0.0823 | 0.0545 |
| 3 | 1.11 | -0.0458 | 0.0280 | 1.07 | -0.0353 | 0.0155 |
| 4 | 0.946 | -0.0293 | 0.0304 | 1.11 | -0.0571 | 0.0195 |

**Ionic Conductivity vs. Compressive Modulus**

In order to check for correlation between ionic conductivity and modulus, Figure S3 plots ionic conductivity vs. linear equivalent modulus for 500 nm and 5 µm LLZTO, before and after cyclic compression (four replicates in each case). The linear equivalent modulus E up to the maximum strain of 30% was calculated based on the strain energy density u, where $E = 2u/\varepsilon^2$ and $u = \int \sigma d\varepsilon$ (computed as the area under the stress-strain curve).

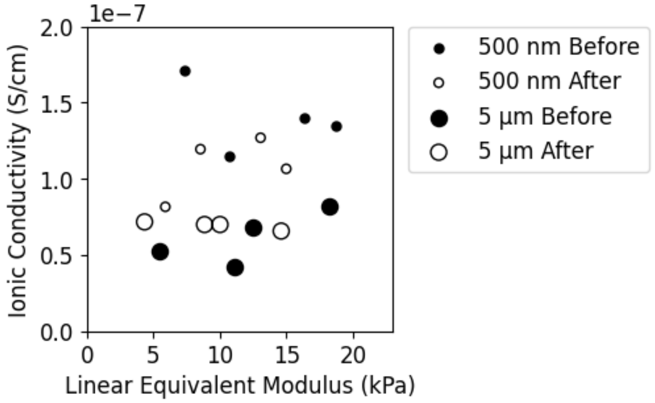


**Figure S3.** Ionic conductivity vs. linear equivalent modulus for electrolytes with 500 nm and 5 µm LLZTO particles ($n = 4$ replicates each). There is no apparent correlation between ionic conductivity and modulus.

**Thickness Before and After Cyclic Compression**

The thickness of each specimen was measured using a digital micrometer before and after cyclic compression. As evidenced in Figure S5, there was no significant change in thickness for both types of CPEs (i.e., those with 500 nm or 5 µm LLZTO particles).

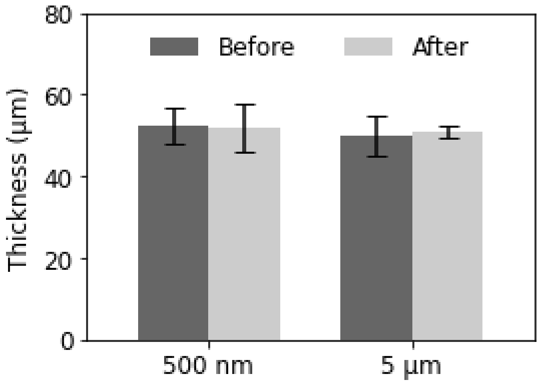


**Figure S4.** Thickness of electrolyte specimens before and after 500 cycles of compression. Column heights show mean thickness values and error bars are shown at ± one standard deviation among $n$ = 4 replicates.

**X-ray Tomography**

The following four 3-D video renderings are provided as electronic files in AVI format. Each video shows an apex corner of the respective type of specimen (500 nm vs. 5 µm particle size, before and after 500 cycles of compression to 30% strain). Each video spins the view, cuts through the volume, and then spins again.

1. 500nm_uncycled.avi
2. 500nm_cycled.avi
3. 5micron_uncycled.avi
4. 5micron_cycled.avi
